# Characterization and Transferable Utility of Microsatellite Markers in the Wild and Cultivated *Arachis* Species

**DOI:** 10.1371/journal.pone.0156633

**Published:** 2016-05-31

**Authors:** Li Huang, Bei Wu, Jiaojiao Zhao, Haitao Li, Weigang Chen, Yanli Zheng, Xiaoping Ren, Yuning Chen, Xiaojing Zhou, Yong Lei, Boshou Liao, Huifang Jiang

**Affiliations:** 1 Key Laboratory of Biology and Genetic Improvement of Oil Crops, Ministry of Agriculture, Oil Crops Research Institute of the Chinese Academy of Agricultural Sciences, Wuhan, Hubei, China; 2 National Key Laboratory of Crop Genetic Improvement, Huazhong Agricultural University, Wuhan, Hubei, China; USDA-ARS-SRRC, UNITED STATES

## Abstract

Microsatellite or simple sequence repeat (SSR) is one of the most widely distributed molecular markers that have been widely utilized to assess genetic diversity and genetic mapping for important traits in plants. However, the understanding of microsatellite characteristics in *Arachis* species and the currently available amount of high-quality SSR markers remain limited. In this study, we identified 16,435 genome survey sequences SSRs (GSS-SSRs) and 40,199 expressed sequence tag SSRs (EST-SSRs) in *Arachis hypogaea* and its wild relative species using the publicly available sequence data. The GSS-SSRs had a density of 159.9–239.8 SSRs/Mb for wild *Arachis* and 1,015.8 SSR/Mb for cultivated *Arachis*, whereas the EST-SSRs had the density of 173.5–384.4 SSR/Mb and 250.9 SSRs/Mb for wild and cultivated *Arachis*, respectively. The trinucleotide SSRs were predominant across *Arachis* species, except that the dinucleotide accounted for most in *A*. *hypogaea* GSSs. From *Arachis* GSS-SSR and EST-SSR sequences, we developed 2,589 novel SSR markers that showed a high polymorphism in six diverse *A*. *hypogaea* accessions. A genetic linkage map that contained 540 novel SSR loci and 105 anchor SSR loci was constructed by case of a recombinant inbred lines F_6_ population. A subset of 82 randomly selected SSR markers were used to screen 39 wild and 22 cultivated *Arachis* accessions, which revealed a high transferability of the novel SSRs across *Arachis* species. Our results provided informative clues to investigate microsatellite patterns across *A*. *hypogaea* and its wild relative species and potentially facilitate the germplasm evaluation and gene mapping in *Arachis* species.

## Introduction

Cultivated peanut (*Arachis hypogaea* L.) is an important oil crop in the world for its direct consumption in the food industry and edible oil in cooking. It is widely cultivated in more than 100 countries with a global annual production of 45.7 Mt over an area of 25.4 Mha (http://faostat.fao.org/faostat/collectons?subset=argriculture 2013). Peanut belongs to the genus *Arachis* containing at least 80 species that are divided into nine taxonomic sections based on morphological variation, geographical distribution and cross-compatibility [1]. All wild *Arachis* species are diploid (2n = 2x = 18, 2n = 2x = 20) except *A*. *monticola* and certain species in section *Rhizomatosae*, whereas peanut is an allotetraploid species (AABB, 2n = 4x = 40) harboring A and B genomes [1]. Cytology and molecular studies have indicated that cultivated peanut results from a single hybridization event between the wild diploid species *A*. *duranensis* (AA, 2n = 2x = 20) and *A*. *ipaënsis* (BB, 2n = 2x = 20) followed by spontaneous chromosome duplication [2, 3].

The studies of genetic diversity in the *Arachis* genus have revealed that cultivated peanut possesses a narrow genetic base compared with wild *Arachis* species [4–7] perhaps due to the bottleneck effect in domestication and little natural gene exchange between wild *Arachis* species and cultivated peanut. Microsatellites, or simple sequence repeats (SSRs), defined as 1–6 nucleotides tandem repeats in the genomes, are mainly caused by replication slippage, leading to addition or removal of repeat motifs in plant genomes. Thus, the certain number and length of SSRs were probable to reflect the evolutionary history in particular species and its relatives [8].

Since no reference genome was currently available in any of *Arachis* species, the SSRs were still the preferable markers in assessing genetic diversity, genetic mapping and marker-assisted selection of important traits as SSRs are multi-allelic, easily detectable by PCR, abundantly distributed in genomes and codominantly inherited [9]. Although the *Arachis* community has made large efforts to develop microsatellite markers [10–18], there was little microsatellite characterization using a large number of sequences in the wild and cultivated *Arachis* species [18] and the understanding of microsatellite characteristics in *Arachis* species remains ambiguous. Furthermore, despite a large number of QTLs identifed for important traits [19–25], the publicly available SSR markers remains insufficient for studies in gene fine-mapping and genome wide association mapping.

Currently, a total of 65,111 genome survey sequences (GSSs) and 281,115 expressed sequence tags (ESTs) of *Arachis* species are publicly available in NCBI (www.ncbi.nlm.nih.gov) [accessed 19 December 2014]. In this study, GSSs and ESTs in *Arachis* species were utilized to perform microsatellite characterization and marker development. The main objectives of this study were: (a) to characterize and compare the frequency, type of microsatellites in the assembled GSSs and ESTs of *Arachis* species; (b) to develop a set of novel SSR markers and validate the reliability and polymorphisms using six *A*. *hypogaea* accessions; (c) to genetically map the novel SSRs on the linkage groups of *A*. *hypogaea* by linkage analysis; (d) to test the utility of the novel SSR markers across the *A*. *hypogaea* and its wild relative species.

## Materials and Methods

### Plant materials and DNA isolation

A set of six diverse peanut accessions (Fuchuan Dahuasheng, ICG6375, Xuhua 13, Zhonghua 6, Zhonghua 10 and ICG12625) were used to screen the polymorphisms of SSR markers. These varieties have been used as parents for three established mapping populations. Fuchuan Dahuasheng (*A*. *hypogaea* var. *hirsuta*), Xu hua 13 (*A*. *hypogaea* var. *hypogaea*) and Zhonghua 10 (*A*. *hypogaea* var. *vulgaris*) are cultivars with large pods and seeds. Zhonghua 6 (*A*. *hypogaea* var. *vulgaris*) is a cultivar with small pods and seeds. ICG6375 (*A*. *hypogaea* var. *vulgaris*) and ICG12625 (*A*. *hypogaea* var. *aequatoriana*) are groundnut varieties received from the International Crop Research Institute for the Semiarid Tropics. ICG6375 has small pods and seeds and ICG12625 has high plant height and dark purple testa. A recombinant inbred line (RIL) F_6_ population (n = 140) derived from the cross by Zhonghua 10 and ICG12625 was used to construct genetic linkage map. Finally, a subset of SSR markers evenly distributed on the linkage map were selected to evaluate the SSR transferability in the different *Arachis* species, containing 39 wild *Arachis* accessions representing five type genomes (A, B, P, E, and AB genome) and 22 cultivated peanut accessions. Detailed information for the 61 *Arachis* accessions was listed in S1 Table.

Genomic DNA was extracted from young leaves of these above mentioned accessions using a modified cetyltrimethyl ammonium bromide (CTAB) method. The integrity and quality of the DNA was evaluated on a 1% agarose gel by comparison with uncut lambda DNA.

### Source of sequences and SSR identification

The GSSs and ESTs of *Arachis* species were downloaded in FASTA format from GenBank and subsequently used for SSR mining and marker development. These sequences downloaded were utilized to identify and select the SSR-containing sequences using SPUTNIK software (http://espressosoftware.com/pages/sputnik.jsp). The criteria for SSR selection were set at six repeats for dinucleotides and four repeats for tri-, tetra- and pentanucleotides. To remove the redundant SSR-containing sequences, the repetitive sequences were masked using RepeatMasker (http://www.repeatmasker.org) and then the masked sequences were assembled using CAP3 software (http://pbil.univ-lyon1.fr/cap.php) with overlap length cutoff of 40 nucleotides and overlap percent identity cutoff of 95. After removing redundancy, the resulting consensus sequences of contigs and singletons were again mined for SSRs.

### Development of SSR marker and polymorphism detection

The above mentioned non-redundant SSR-containing sequences were employed to design PCR primers using Primer3 software [26]. The primer length was between 18 and 23 nucleotides with an optimum size of 20 nucleotides. The melting temperatures ranged from 50 to 70°C with an optimum temperature of 55°C. The GC content varied from 30% to 70% with an optimum GC content of 50%. The predicted PCR products ranged from 100 to 400 bp. After filtering out the SSRs with identical primer sequence to the publicly released SSRs in *Arachis* species, the novel SSR markers were identified in the present study and designated as “AGGS”, representing *A**rachis* genus GenBank sequence.

The novel SSRs were tested for the reliability and polymorphism using a set of six diverse peanut accessions mentioned above. PCR amplifications were performed in a volume of 10 μl including 50 ng genomic DNA, 1 × Taq buffer, 2 mM MgCl_2_, 0.2 mM dNTPs, 0.2 μM each primer and 0.25 U Taq DNA polymerase. Then the amplification was conducted by the ‘touchdown’ method, with the following thermal profile: initial denaturation at 94°C for 5 min; ten cycles of 30 s at 94°C, 30 s at 65°C with a 1°C decrease in annealing temperature per cycle and 45 s at 72°C; 30 cycles of 30 s at 94°C, 30 s at 55°C and 45 s at 72°C and a final extension at 72°C for 10 min. The PCR products were visualized on 6% polyacrylamide gel followed by silver staining. The fragment sizes of PCR products were estimated by comparison with a 50 bp DNA ladder.

### Linkage analysis and map construction

The recombinant inbred lines (RIL) F_6_ population containing 140 individuals derived from the cross between Zhonghua 10 and ICG12625 were developed in our laboratory. The polymorphic SSR markers between Zhonghua 10 and ICG12625 were used to construct a genetic linkage map. For each SSR marker, the set of the amplified SSR alleles (or fragments) were classified into a single-locus SSR, if they exhibited an obvious co-dominant pattern across the whole population and the observed heterozygosity rate was less than 5% as described previously [27, 28]. These multi-locus SSRs were named using the suffixes “-1”, “-2” and “-n” after the SSR name, respectively. For example, the locus AGGS0010-3 meant the third locus amplified by the SSR marker AGGS0010. The marker loci were grouped at logarithm of odds (LOD) 4.0 by JoinMap 3.0 [29]. The Kosambi mapping function was used to convert the recombination frequency into genetic distance [30]. Pearson’s Chi square test was performed to evaluate the goodness of fit to the expected 1:1 segregation ratio for each marker. In order to assign the novel SSR markers to specific linkage groups, a set of 105 public SSRs that were previously evenly mapped on A1-A10 and B1-B10 [24] were used as anchor markers.

### Evaluation of the SSR markers across *Arachis* species

To test the transferability of the novel SSR markers in different *Arachis* species and evaluate the utility in genetic diversity and phylogenic analysis, a set of 82 SSR markers that contained 19 SSRs originated from the A-genome species, 22 SSRs from the B-genome species and 41 SSRs from the AB-genome species were selected to genotype 39 wild *Arachis* accessions and 22 cultivated peanut accessions. The allele richness for SSR markers were estimated for the wild and cultivated *Arachis* accessions using the PowerMarker v3.51 package [31]. The Nei’s distances [32] between all pairs of accessions were calculated and a phylogenic tree was constructed with the Neighbor-joining (N-J) algorithm to depict the genetic relationship and differentiation between the wild and cultivated *Arachis* accessions [33].

## Results

### Identification and characterization of microsatellites from GSSs in *Arachis* species

A total of 65,111 GSSs available of *Arachis* spp. were obtained in the GenBank database (accessed 19 December 2014), including 44,761 GSSs from *A*. *duranensis* (AA genome), 3,276 GSSs from *A*. *batizocoi* (BB genome) and 17,074 GSSs from *A*. *hypogaea* (AABB genome), respectively. Through searching the di-, tri-, tetra-, and pentanucleotide SSR repeats in GSS sequence, a total of 4,401, 377 and 7,251 SSR-containing sequences were identified in *A*. *duranensis*, *A*. *batizocoi* and *A*. *hypogaea*, respectively (Table 1). All these SSR containing sequences were aligned with each other to remove the redundant GSSs, resulting in a total of 445 contigs and 10,823 singletons. Interestingly, a large fraction of singletons were identified in either cultivated *Arachis* species (6,888/7,251; *A*. *hypogaea*) or the two wild *Arachis* species (i.e., 3,574/4,401 for *A*. *duranensis* and 361/377 for *A*. *batizocoi*), indicating that the public data is informative and of little redundancy. By integrating the contigs and singletons, we finally identified 4,826, 454 and 11,155 unique GSS-SSRs with an overall frequency of 159.9, 239.8 and 1,015.8 SSRs/Mb in *A*. *duranensis*, *A*. *batizocoi* and *A*. *hypogaea*, respectively (Table 1).

**Table 1:** Information of sequences containing repeats in *Arachis* species.

Sequences type	Species	Genome	No. of sequences surveyed	Length of sequences searched (bp)	No. of sequences containing repeats	Singletons No.	Contigs No.	No. of sequences per contig	No. of sequences containing repeats after removing redundant sequences	No. of SSRs after removing redundant sequences	Overall density (SSRs/Mb)
GSS	*A*. *duranensis*	A	44,761	30,185,265	4,401	3,574	263	3	4,072	4,826	159.9
	*A*. *batizocoi*	B	3,276	1,892,928	377	361	8	2	377	454	239.8
	*A*. *hypogaea*	AB	17,074	10,981,790	7,251	6,888	174	2	7,194	11,155	1,015.8
EST	*A*. *appressipila*	P	400	216,494	62	56	3	2	60	69	318.7
	*A*. *duranensis*	A	35,292	17,417,205	5,980	2,870	832	4	4,605	5,857	336.3
	*A*. *stenosperma*	A	6,264	4,213,240	868	689	67	3	805	969	230.0
	*A*. *ipaënsis*	B	32,787	14,420,631	5,694	2,883	764	4	4,423	5,543	384.4
	*A*. *magna*	B	750	236,255	77	10	12	5	35	41	173.5
	*A*. *hypogaea*	AB	205,622	110,480,671	33,286	13,795	3,145	6	21,524	27,720	250.9

**Fig 1:**
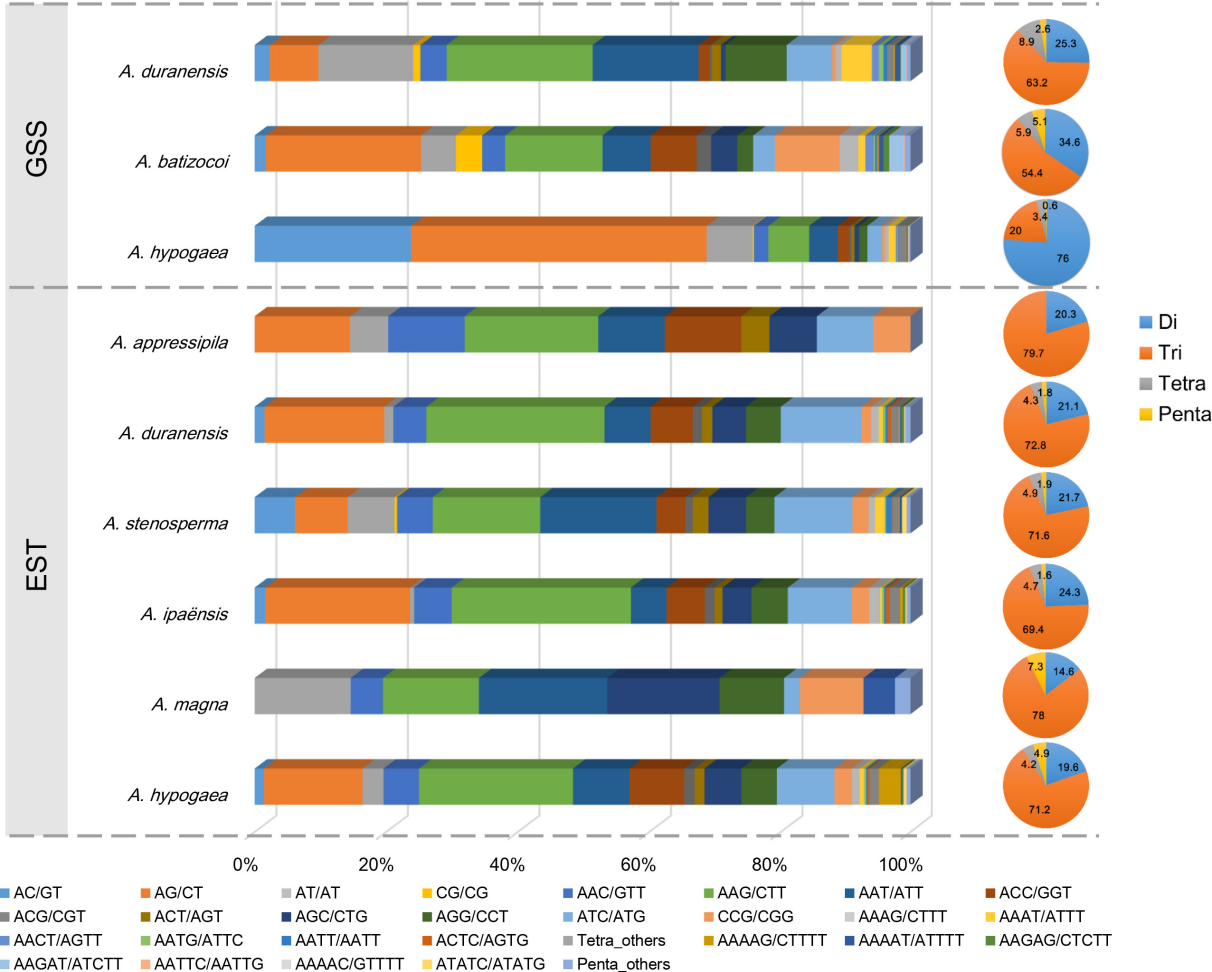
Distribution of the motif and repeat type of microsatellites in the assembled GSSs and ESTs in *Arachis* species. For the bar plot, the horizontal axis showed the abundances (%) of microsatellites with different motif types (the legends lay below the plot). For the pie plots, the portions with different colors showed the proportions (%) of microsatellites with different repeat types (the legends lay right of the plot). In the plot, only the repeat and motifs from dinucleotide to pentanucleotide SSRs were shown.

Of the total GSS-SSRs identified in the three species, the di- and trinucleotide repeat motifs accounted for nearly 90% (Fig 1, S2 Table). The trinucleotide repeat motifs were the most abundant repeat types in *A*. *duranensis* and *A*. *batizocoi*, while the dinucleotide repeat motifs were the most abundant in *A*. *hypogaea*. Tetra- and pentanucleotide repeat motifs both had a low frequency (0.6~8.9%) in all the three species. In dinucleotide repeat motifs, the most abundant repeat motif were (AG/CT)_n_ in *A*. *batizocoi* (23.6%) and *A*. *hypogaea* (45.0%), while (AT/AT)_n_ (14.4%) in *A*. *duranensis* (Fig 1, S2 Table). All ten possible combinations of trinucleotide repeat motifs were observed in the three species except in *A*. *batizocoi*. Among these trinucleotide repeat motifs, (AAG/CTT)_n_ was the most common motif in *A*. *duranensis*, *A*. *batizocoi* and *A*. *hypogaea*, with the frequency of 22.2%, 14.8% and 6.2%, respectively (Fig 1, S2 Table).

### Identification and characterization of microsatellites from ESTs in *Arachis* species

In the GenBank database 281,115 ESTs were acquired in *Arachis* spp., which contained 400 ESTs from *A*. *appressipila* (PP genome), 35,292 ESTs from *A*. *duranensis* (AA genome), 6,264 ESTs from *A*. *stenosperma* (AA genome), 32,787 ESTs from *A*. *ipaensis* (BB genome), 750 ESTs from *A*. *magna* (BB genome) and 205,622 ESTs from *A*. *hypogaea* (AABB genome). A total of 62, 5,980, 868, 5,694, 77 and 33,286 SSR-containing sequences were identified from ESTs in *A*. *appressipila*, *A*. *duranensis*, *A*. *stenosperma*, *A*. *ipaensis*, *A*. *magna* and *A*. *hypogaea*, respectively. After removing the redundant sequences, 60, 4,605, 805, 4,423, 35 and 21,524 unique SSR-containing sequences were obtained from ESTs in *A*. *appressipila*, *A*. *duranensis*, *A*. *stenosperma*, *A*. *ipaensis*, *A*. *magna* and *A*. *hypogaea*, respectively. Eventually, 69, 5,857, 969, 5,543, 41 and 27,720 EST-SSRs were identified from these unique SSR-containing sequences in *A*. *appressipila*, *A*. *duranensis*, *A*. *stenosperma*, *A*. *ipaensis*, *A*. *magna* and *A*. *hypogaea*, respectively, with an overall frequency of 318.7, 336.3, 230.0, 384.4, 173.5 and 250.9 SSRs/Mb (Table 1).

Among these EST-SSRs identified, the trinucleotide repeat was the most abundant repeat type in all six *Arachis* species and had a high frequency of 73.82% on average with a range from 69.4% in *A*. *ipaensis* to 79.7% in *A*. *appressipila* (Fig 1, S3 Table). The dinucleotide repeat motif occurred at a median frequency of 20.3% on average with a range from 14.6% in *A*. *magna* to 24.3% in *A*. *ipaensis*. Tetra- and pentanucleotide repeat motifs were observed with a low frequency (0~4.9%) in the six *Arachis* species. Among the dinucleotide repeats, (AG/CT)_n_ motif was observed with the highest frequency in the six *Arachis* species except *A*. *magna*, in which only one dinucleotide type (AT/AT)_n_ was observed. All ten possible combinations of trinucleotide repeat motifs were identified in the six *Arachis* species except *A*. *appressipila* and *A*. *magna*, possibly due to the significantly less available ESTs. Among these trinucleotide repeats, the most abundant motif was (AAG/CTT)_n_ for *A*. *appressipila*, *A*. *duranensis*, *A*. *ipaensis* and *A*. *hypogaea*, while (AAT/ATT)_n_ for *A*. *stenosperma* and *A*. *magna* (Fig 1, S3 Table).

### Development of SSR markers and polymorphism analysis

**Fig 2:**
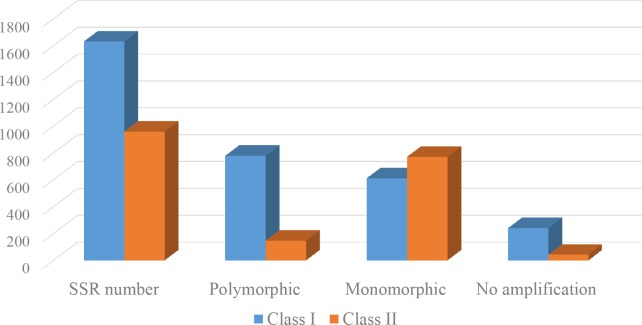
The distribution of novel SSRs polymorphic in six *A*. *hypogaea* cultivars.

After filtering out the SSRs with identical primer sequence to the publicly released SSRs in *Arachis* species, we totally identified 2,589 novel SSR markers in the present study (S4 Table). The set of novel SSRs from *Arachis* species were tested for reliability and polymorphism by amplification in six *A*. *hypogaea* accessions. Among 2,589 novel SSRs, there were 2,207 SSRs (85.2%) that the primers enable to amplify one or more clear fragments and 925 SSRs (35.7%) showing polymorphism in the six *A*. *hypogaea* accessions. The dinucleotide repeat (39.7%) and the (AG/CT)_n_ motif (42.0%) accounted for the largest fraction of the novel SSRs, and these SSRs also exhibited the highest level of polymorphism in *A*. *hypogaea* (S5 Table). To further distinguish the characteristic of SSR markers, the whole set of developed SSRs were classified into two major classes based on the length of SSR. The ‘Class I’ type of SSRs were defined as the ones with the length of SSR more than 20 bp, and the ‘Class II’ type of SSRs as the ones with the length of SSR less than 20 bp but more than 12 bp [34]. The ‘Class I’ type of SSRs contained 959 SSRs and the ‘Class II’ type of SSRs contained 1,630 SSRs. Notably, there were 47.8% of SSRs as ‘Class I’ type while 15.2% of SSRs as ‘Class II’ type that were polymorphic in the six accessions, respectively (Fig 2).

### Construction of the genetic linkage map

**Fig 3:**
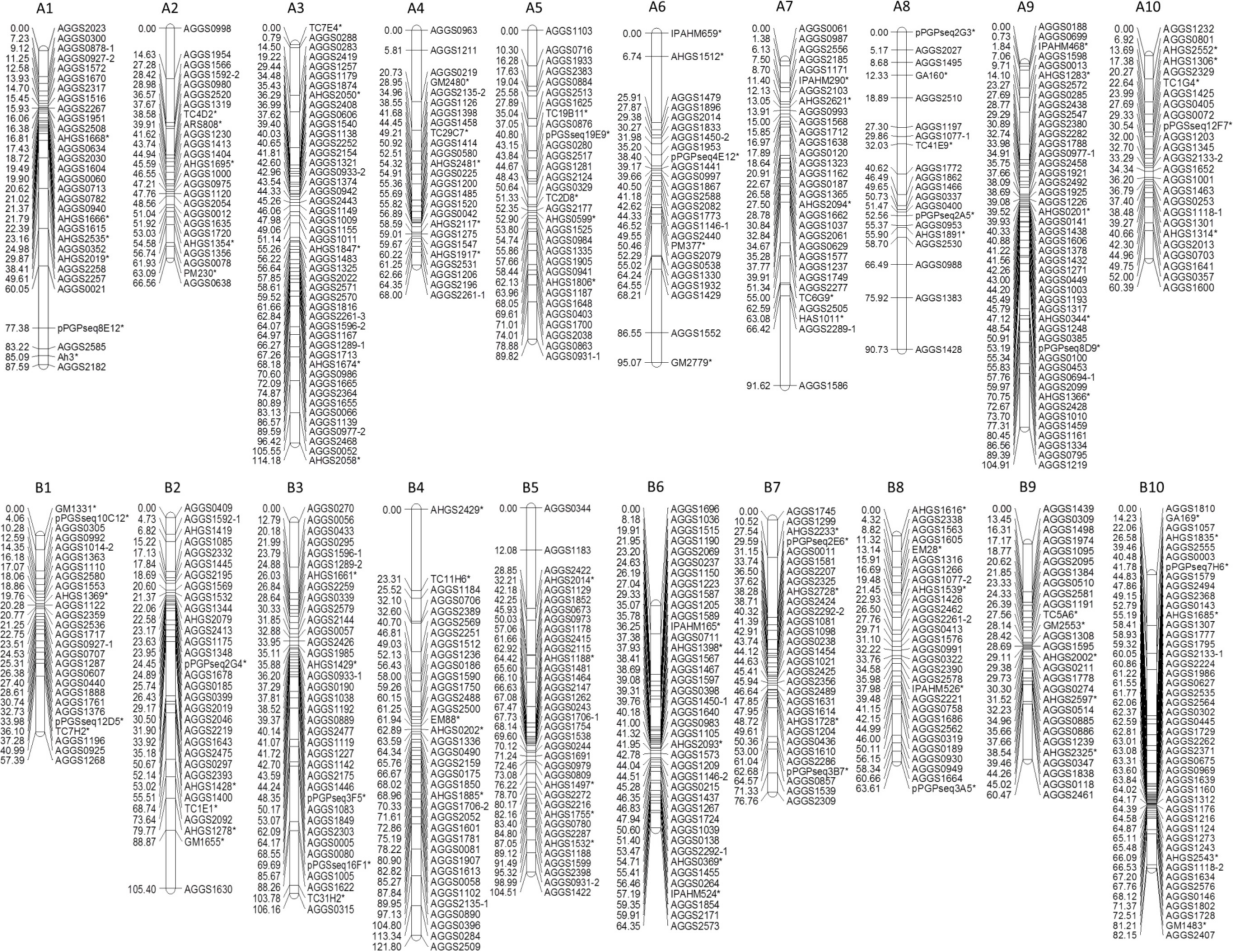
The genetic linkage map of *Arachis hypogaea* with the novel SSRs developed in present study. The linkage map were constructed in a RIL population from Zhonghua 10 × ICG12625. The SSR loci with suffix “*” were anchor loci that were selected from a previously published linkage map (Huang et al. 2015).

Among the 925 polymorphic SSRs in six diverse *A*. *hypogaea* accessions, a subset of 559 SSR markers that were polymorphic between Zhonghua 10 and ICG12625 were employed to genotype a derived RIL population for allocating the specific positions to the novel SSRs by genetic linkage map. According to segregation patterns of SSRs, the 559 polymorphic SSRs totally identified 580 SSR loci in the RIL population. There were one SSR with three loci, 19 SSRs with two loci for each SSR and 539 SSRs with a single-locus for each SSR. With the set of 580 novel SSR loci and 105 anchor SSR markers, a genetic linkage map consisting of 20 linkage groups was constructed (Fig 3). The genetic map was covered by a total of 645 SSR loci with the total length of 1,711.47 cM and an average interval of 2.65 cM between flanking markers (S6 Table). The 20 linkage groups were designated as A1-A10 for the A subgenome and B1-B10 for the B subgenome by aligning anchor markers to a previously published map [24].

In the linkage map, there were totally 305 SSR loci for the A subgenome with 253 novel SSR loci and 52 anchor loci, while 340 SSR loci for the B subgenome with 287 novel SSR loci and 53 anchor loci (Table 2). All of the anchor SSRs were mapped to the originally reported linkage groups [24], indicating the reliability of linkage map constructed using these novel SSR markers. In the A subgenome, the number of novel SSR loci on the linkage groups varied from 41 (A3) to 15 (A8) with an average of 25. In the B subgenome, all the linkage groups contained more than 22 novel SSR loci, among which the B10 linkage group had the most novel SSR loci (39). In the linkage map, Chi square (χ^2^) analysis revealed that only 57 of 580 novel SSR loci exhibited a significant level of segregation distortion in the RIL population (*P*<0.05) (S6 Table), which further verified the reliability of the novel SSRs in present study.

**Table 2:** Information of linkage group constructed using the novel SSRs in present study.

Linkage Group	SSR loci	Anchor Loci	Novel Loci	Length (cM)
A1	31	6	25	87.59
A2	25	5	20	66.56
A3	46	5	41	114.18
A4	25	5	20	68.00
A5	31	5	26	89.82
A6	25	5	20	95.07
A7	31	5	26	91.62
A8	20	5	15	90.73
A9	46	6	40	104.91
A10	25	5	20	60.39
B1	27	5	22	57.39
B2	32	7	25	105.40
B3	37	5	32	106.16
B4	36	5	31	121.80
B5	36	5	31	104.51
B6	40	5	35	64.35
B7	30	5	25	76.76
B8	29	5	24	63.61
B9	28	5	23	60.47
B10	45	6	39	82.15
A subgenome	305	52	253	868.87
B subgenome	340	53	287	842.60
whole genome	645	105	540	1,711.47

### The utility of novel SSR markers in *A*. *hypogaea* and its wild related species

**Fig 4:**
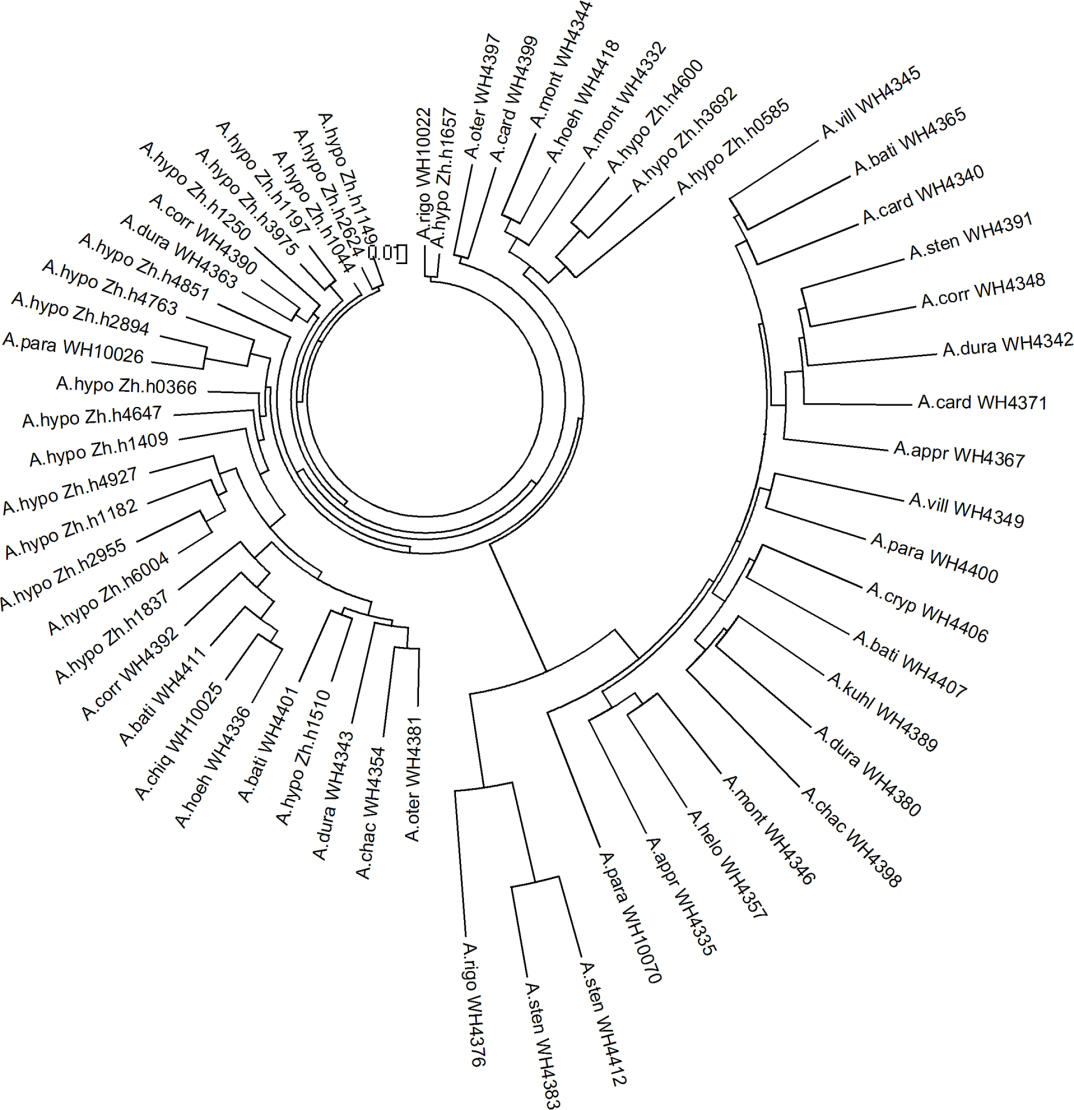
The phylogenic analysis of the 61cultivated and wild *Arachis* accessions in this study.

To test the transferability of the novel SSR markers in different *Arachis* species, we randomly selected 82 SSRs from linkage map to genotype 22 *A*. *hypogaea* accessions and 39 of its wild relatives. Among these SSRs, there were 37 SSRs that successfully amplified all 61 tested *Arachis* accessions, including 9 SSRs (47.4%) derived from *A*. *duranensis*, 4 SSRs (18.2%) from *A*. *batizocoi* and 24 SSRs (58.5%) from *A*. *hypogaea* (Table 3). Notably, all 82 SSRs not only amplified clear fragments in each of 22 *A*. *hypogaea* accessions, but also exhibited a relatively high amplification rate in the wild *Arachis* accessions for AB genome (77.3~97.6%), B genome (81.8~85.4%) and E genome (81.8~85.4%). Comparatively, the SSRs derived from *A*. *batizocoi* had a relatively low amplification rate in the wild *Arachis* accessions of P genome (36.4%) and A genome (36.4%) (Table 3). These results revealed that the SSRs derived from *A*. *hypogaea and A*. *duranensis* had relatively higher transferability across the cultivated and wild *Arachis* species than those from *A*. *batizocoi*. Furthermore, it was found that the SSRs from *A*. *hypogaea* had a high level of polymorphism in all accessions with 7.6 alleles/SSR, followed by SSRs from *A*. *batizocoi* (5.1 alleles/SSR) and *A*. *duranensis* (4.6 alleles/SSR). All the SSRs had a similar level of polymorphism in wild *Arachis* accessions of P genome, E genome, A genome, B genome and AB genome, respectively, while the SSRs developed from *A*. *hypogaea* sequences had a significantly higher level of polymorphism in the *A*. *hypogaea* accessions than those SSRs developed from *A*. *duranensis* sequences and *A*. *batizocoi* sequences (Table 3). Additionally, the novel SSRs were used to evaluate the genetic relationship among the 61 wild and cultivated *Arachis* accessions. A phylogenic dendrogram based on the N-J algorithm was constructed that clustered all the *Arachis* accessions into two major genetic groups basically in accordance to the *Arachis* taxonomy (Fig 4). The largest group consisted of all the *A*. *hypogaea* accessions and the majority of accessions from its diploid ancestor of A and B genomes. The diploid and tetrapolyploid wild *Arachis* accessions were clustered into a separate large group. Our results indicated that the novel SSRs had a high transferability across the *Arachis* species and had the ability to assess genetic diversity and phylogenic relationship between wild and cultivated *Arachis*.

**Table 3:** The amplification of SSRs derived from *A*. *duranensis*, *A*. *batizocoi* and *A*. *hypogaea* in *Arachis* species.

SSR resources	SSRs No.	Amplified SSRs (%)							alleles/SSR						
		I	II	III	IV	V	VI	VII	I	II	III	IV	V	VI	VII
*A*. *duranensis*	19	9 (47.4)	10 (52.6)	15 (78.9)	11 (57.9)	15 (78.9)	17 (89.5)	19 (100)	4.6	3.1	2.8	4.3	2.6	2.3	2.4
*A*. *batizocoi*	22	4 (18.2)	8 (36.4)	18 (81.8)	8 (36.4)	18 (81.8)	17 (77.3)	22 (100)	5.1	3.3	3	4.5	2.9	2.6	1.7
*A*. *hypogaea*	41	24 (58.5)	30 (73.2)	35 (85.4)	27 (65.9)	35 (85.4)	40 (97.6)	41 (100)	7.6	2.8	3	5	3.3	2	4.9

I: all *Arachis* accessions; II: 5 wild *Arachis* accessions of P genome; III: 6 wild *Arachis* accessions of E genome; IV: 19 wild *Arachis* accessions of A genome; V: 6 wild *Arachis* accessions of B genome; VI: 3 wild *Arachis* accessions of AB genome; VII: 22 *A*. *hypogaea* accessions

## Discussion

To date, there was little knowledge about microsatellite distributions in the wild and cultivated *Arachis* species. In this study, the microsatellite characteristics across the *Arachis* species were analyzed by using the publicly released GSS and EST sequences. We found that the GSSs had a SSR density of 159.9 and 239.8 SSRs/Mb for two diploid wild *Arachis* species (*A*. *duranensis* and *A*. *batizocoi*), but exhibited an extremely high density of 1,025.8 SSRs/Mb for the tetrapolyploid *Arachis* (*A*. *hypogaea*). The microsatellite density in *Arachis* species was sharply higher than those in *Gossypium* species (41.2 to 49.1 SSRs/Mb) [35], but lower than those in *Brassica* crop species (420.6 to 496.8 SSRs/Mb) [36] except in *A*. *hypogaea*. The significantly higher SSR density of *A*. *hypogaea* relative to other *Arachis* species may be attributed to the lack and the uneven genomic distribution of GSS sequences in the *Arachis* species. In the present study, the length of assessed sequences accounted for a small proportion of genome in each *Arachis* species, which might lead to the difficulties for accurately evaluating characteristics of microsatellite across the *Arachis* species. On the other hand, some of the sequences of GenBank database were obtained with the initial focus on mining and development of microsatellite markers in the previous studies, thus probably resulting in overestimating the numbers and density of SSRs in the present study, especially in *A*. *hypogaea* [11, 13, 16]. For ESTs, the tetrapolyploid *Arachis* species (*A*. *hypogaea*) had a SSR density of 250.9 SSRs/Mb, but its diploid wild ancestors (*A*. *duranensis* and *A*. *ipaensis*) had a relatively high SSR density of 336.3 and 384.4 SSRs/Mb. This result was congruent to the finding that the genetic diversity of cultivated peanut was lower than the wild diploid *Arachis* species [7], which may be attributed to the origin of cultivated peanut in a single allopolyploid event between the wild diploid ancestors, *A*. *duranensis* and *A*. *ipaensis* [2, 3]. Despite the limitation of the current data, the results of this study provided an informative clue to characterize the differentiation of microsatellites across the *Arachis* species. With the future release of the reference genomes of the cultivated peanut and its two diploid ancestors, it would be possible to comprehensively characterize the evolutionary pattern of microsatellites between wild and cultivated *Arachis* species.

The composition of microsatellites varied across the *Arachis* species. In GSSs, the dinucleotide repeats dominated in *A*. *hypogaea* (76.0%), which is similar to Chinese spring wheat [8], rice and *Arabidopsis* [37], while the trinucleotide repeats were the predominant in *A*. *duranensis* and *A*. *batizocoi* (63.2% and 54.4%) as well as *Brachkypodium* [38], bamboo [39] and *Setaria italica* [40]. These results revealed the different patterns of microsatellite motifs across the plant species, which may reflected the genomic footprints of speciation and evolution in these species. In ESTs, the trinucleotide repeats were predominant across all the *Arachis* species. This result was in accordance with previous ones on EST-SSR in peanut [18]. However, it was inconsistent with another study on GSS-SSR and EST-SSR, in which 3.5 Mbp cultivated peanut genome sequences and 29.3 Mbp *A*. *duranensis* genome sequences were used to identify microsatellites [17]. This may be attributed to the data from the different *Arachis* species used in these studies. The use of more *Arachis* species in present study would be more reliable to infer the pattern of microsatellites in *Arachis* genus, although the large amount of data and reference genome of the *Arachis* species were still needed to make a comprehensive evolutionary conclusion in *Arachis* genus. In the present study, the most frequent dinucleotide repeat motif was (AG/CT)_n_, which was consistent with that identified in the previous studies in peanut [10, 11, 14, 16, 18], while the dinucleotide motif (AT)_n_ was enriched in Chinese spring wheat [8], *Gossypium* species [35] and *Brassica* crop species [36, 41, 42]. Additionally, the top two trinucleotide repeat motifs were (AAG/CTT)_n_ and (AAT/ATT)_n_ in this study, which was in accordance with those results of previous studies in peanut [10, 11, 14, 16, 18], *Gossypium* species [35] and *Brassica* crop species [36, 41, 42].

In *Arachis*, SSRs has served as an informative and manageable marker in germplasm evaluation, QTL analysis and marker-assisted selection for a long time. Extensive efforts had been made to develop SSR markers in *Arachis* from various resources [10–18], but the amount of SSR markers remain insufficient to map QTL in fine-scale or saturate the whole genome for genome wide association study (GWAS) [23–25, 43–45], due to the low genetic diversity in *A*. *hypogaea*. To this end, we developed novel 2,589 SSRs based on the publicly released *Arachis* sequences in present study. The majority of the novel SSRs were able to clearly amplify fragments in *A*. *hypogaea*, and 540 of these markers were successfully allocated to the specific genetic positions in linkage groups of *A*. *hypogaea*. These results would provide an informative resource to localize QTL and marker-assisted selection. Moreover, as the polyploidy nature of *A*. *hypogaea*, the SSRs probably amplify multiple alleles at homologous DNA sequences. That fact would lead to a substantial risk of allele calling bias in natural populations for GWAS in tetrapolyploid species [27, 28, 46]. Therefore, there is an urgent need to develop the single-locus SSR markers to facilitate their applications, as currently only a small proportion of the previously developed publicly available SSRs were single-locus markers [43–45]. In the present study, we totally identified 539 SSRs that were probably to be single-locus markers according to the segregation pattern in a biparental RIL population. These single-locus SSRs with the specific genetic positions gave the research community a useful resource to evaluate linkage disequilibrium in *Arachis*, although they were still needed to be validated by different mapping populations. Additionally, the novel SSRs in this study were highly transferable across *Arachis* species, especially for *A*. *hypogaea*, and would be informative to assess genetic diversity and phylogenic relationship between wild and cultivated *Arachis*.

## Supporting Information

S1 Table Information for the 61 cultivated and wild *Arachis* accessions used in this study. (XLSX)

S2 Table Number, repeat number and total repeat length of the di- to pentanucleotide repeats motifs of microsatellites in the assembled GSSs of *Arachis* species. (XLSX)

S3 Table Number, repeat number and total repeat length of the di- to pentanucleotide repeats motifs of microsatellites in the assembled ESTs of *Arachis* species. (XLSX)

S4 Table Detailed information for the novel SSRs developed in the present study. (XLSX)

S5 Table Polymorphism rates of different repeat motifs in six *A*. *hypogaea* cultivars. (XLSX)

S6 Table The detailed information of genetic positions and segregation distortion of the SSRs in the linkage map. (XLSX)

## Abbreviations

BAC: bacterial artificial chromosome
CTAB: cetyltrimethyl ammonium bromide
EST: expressed sequence tag
GSS: genome survey sequence
GWAS: genome wide association study
LG: linkage group
LOD: logarithm of odds
PCR: polymerase chain reaction
RIL: recombinant inbred lines
SSR: simple sequence repeat

## References

[1] Krapovickas A, Gregory WC. Taxonomía del género *Arachis* (Leguminosae). Bonplandia. 1994;8(1–4):1–186.

[2] Kochert G, Stalker HT, Gimenes M, Galgaro L, Lopes CR, Moore K. RFLP and cytogenetic evidence on the origin and evolution of allotetraploid domesticated peanut, *Arachis hypogaea* (Leguminosae). Am J Bot. 1996;83:1282–91.

[3] Seijo J, Lavia G, Fernández A, Krapovickas A, Ducasse D, Moscone E. Physical mapping of 5S and 18S-25S rRNA genes evidences that *Arachis duranensis* and *A*. *ipaënsis* are the wild diploid species involved in the origin of *A*. *hypogaea* (Leguminosae). Am J Bot. 2004;91:1294–303. 10.3732/ajb.91.9.1294 21652361

[4] Halward TM, Stalker HT, Larue EA, Kochert G. Genetic variation detectable with molecular markers among unadapted germplasm resources of cultivated peanut and related wild species. Genome. 1991;34(6):1013–20.

[5] Kochert G, Halward T, Branch W, Simpson C. RFLP variability in peanut (*Arachis hypogaea* L.) cultivars and wild species. Theor Appl Genet. 1991;81(5):565–70. 10.1007/BF00226719 24221368

[6] He G, Prakash CS. Identification of polymorphic DNA markers in cultivated peanut (*Arachis hypogaea* L.). Euphytica. 1997;97(2):143–9.

[7] Subramanian V, Gurtu S, Rao RN, Nigam S. Identification of DNA polymorphism in cultivated groundnut using random amplified polymorphic DNA (RAPD) assay. Genome. 2000;43(4):656–60. 10984178

[8] Han B, Wang C, Tang Z, Ren Y, Li Y, Zhang D, et al Genome-Wide Analysis of Microsatellite Markers Based on Sequenced Database in Chinese Spring Wheat (*Triticum aestivum* L.). PLoS ONE. 2015;10(11):e0141540 10.1371/journal.pone.0141540 26536014 PMC4633229

[9] Gupta PK, Varshney R. The development and use of microsatellite markers for genetic analysis and plant breeding with emphasis on bread wheat. Euphytica. 2000;113(3):163–85.

[10] He G, Meng R, Newman M, Gao G, Pittman R, Prakash CS. Microsatellites as DNA markers in cultivated peanut (*Arachis hypogaea* L.). BMC Plant Biol. 2003;3(1):3. 12713672 10.1186/1471-2229-3-3 PMC155651

[11] Ferguson M, Burow M, Schulze S, Bramel P, Paterson A, Kresovich S, et al Microsatellite identification and characterization in peanut (*A*. *hypogaea* L.). Theor Appl Genet. 2004;108(6):1064–70. 15067392 10.1007/s00122-003-1535-2

[12] Proite K, Leal-Bertioli SCM, Bertioli DJ, Moretzsohn MC, Da Silva FR, Martins NF, et al ESTs from a wild *Arachis* species for gene discovery and marker development. BMC Plant Biol. 2007;7(1):7. 17302987 10.1186/1471-2229-7-7 PMC1808460

[13] Cuc LM, Mace ES, Crouch JH, Quang VD, Long TD, Varshney RK. Isolation and characterization of novel microsatellite markers and their application for diversity assessment in cultivated groundnut (*Arachis hypogaea*). BMC Plant Biol. 2008;8(1):1–11. 18482440 10.1186/1471-2229-8-55 PMC2416452

[14] Liang X, Chen X, Hong Y, Liu H, Zhou G, Li S, et al Utility of EST-derived SSR in cultivated peanut (*Arachis hypogaea* L.) and *Arachis* wild species. BMC Plant Biol. 2009;9(1):35. 19309524 10.1186/1471-2229-9-35 PMC2678122

[15] Koilkonda P, Sato S, Tabata S, Shirasawa K, Hirakawa H, Sakai H, et al Large-scale development of expressed sequence tag-derived simple sequence repeat markers and diversity analysis in *Arachis* spp. Mol Breeding. 2012;30(1):125–38. 10.1007/s11032-011-9604-8 PMC3362703 22707912

[16] Shirasawa K, Koilkonda P, Aoki K, Hirakawa H, Tabata S, Watanabe M, et al *In silico* polymorphism analysis for the development of simple sequence repeat and transposon markers and construction of linkage map in cultivated peanut. BMC Plant Biol. 2012;12:80 10.1186/1471-2229-12-80 22672714 PMC3404960

[17] Wang H, Penmetsa RV, Yuan M, Gong L, Zhao Y, Guo B, et al Development and characterization of BAC-end sequence derived SSRs, and their incorporation into a new higher density genetic map for cultivated peanut (*Arachis hypogaea* L.). BMC Plant Biol. 2012;12(1):10. 22260238 10.1186/1471-2229-12-10 PMC3298471

[18] Bosamia TC, Mishra GP, Thankappan R, Dobaria JR. Novel and Stress Relevant EST Derived SSR Markers Developed and Validated in Peanut. PLoS ONE. 2015;10(6):e0129127 10.1371/journal.pone.0129127 26046991 PMC4457858

[19] Khedikar Y, Gowda MVC, Sarvamangala C, Patgar K, Upadhyaya H, Varshney R. A QTL study on late leaf spot and rust revealed one major QTL for molecular breeding for rust resistance in groundnut (*Arachis hypogaea* L.). Theor Appl Genet. 2010;121(5):971–84. 10.1007/s00122-010-1366-x 20526757 PMC2921499

[20] Ravi K, Vadez V, Isobe S, Mir R, Guo Y, Nigam S, et al Identification of several small main-effect QTLs and a large number of epistatic QTLs for drought tolerance related traits in groundnut (*Arachis hypogaea* L.). Theor Appl Genet. 2011;122(6):1119–32. 10.1007/s00122-010-1517-0 21191568 PMC3057011

[21] Gautami B, Pandey M, Vadez V, Nigam S, Ratnakumar P, Krishnamurthy L, et al Quantitative trait locus analysis and construction of consensus genetic map for drought tolerance traits based on three recombinant inbred line populations in cultivated groundnut (*Arachis hypogaea* L.). Mol Breeding. 2012;30(2):757–72. 10.1007/s11032-011-9660-0 PMC3410028 22924017

[22] Sujay V, Gowda M, Pandey M, Bhat R, Khedikar Y, Nadaf H, et al Quantitative trait locus analysis and construction of consensus genetic map for foliar disease resistance based on two recombinant inbred line populations in cultivated groundnut (*Arachis hypogaea* L.). Mol Breeding. 2012;30(2):773–88. 10.1007/s11032-011-9661-z PMC3410029 22924018

[23] Pandey MK, Wang ML, Qiao L, Feng S, Khera P, Wang H, et al Identification of QTLs associated with oil content and mapping *FAD2* genes and their relative contribution to oil quality in peanut (*Arachis hypogaea* L.). BMC Genet. 2014;15(1):133. 25491595 10.1186/s12863-014-0133-4 PMC4278341

[24] Huang L, He H, Chen W, Ren X, Chen Y, Zhou X, et al Quantitative trait locus analysis of agronomic and quality-related traits in cultivated peanut (*Arachis hypogaea* L.). Theor Appl Genet. 2015;128(6):1103–15. 10.1007/s00122-015-2493-1 25805315 PMC4434864

[25] Wang ML, Khera P, Pandey MK, Wang H, Qiao L, Feng S, et al Genetic mapping of QTLs controlling fatty acids provided insights into the genetic control of fatty acid synthesis pathway in peanut (*Arachis hypogaea* L.). PLoS ONE. 2015;10:e0119454 10.1371/journal.pone.0119454 25849082 PMC4388682

[26] Rozen S, Skaletsky H. Primer3 on the WWW for general users and for biologist programmers. Methods Mol Biol. 2000;132:365–86. 10547847 10.1385/1-59259-192-2:365

[27] Xiao Y, Cai D, Yang W, Ye W, Younas M, Wu J, et al Genetic structure and linkage disequilibrium pattern of a rapeseed (*Brassica napus* L.) association mapping panel revealed by microsatellites. Theor Appl Genet. 2012;125:437–47. 10.1007/s00122-012-1843-5 22437490

[28] Wu Z, Wang B, Chen X, Wu J, King GJ, Xiao Y, et al Evaluation of linkage disequilibrium pattern and association study on seed oil content in *Brassica napus* using ddRAD sequencing. PLoS ONE. 2016;11:e0146383 10.1371/journal.pone.0146383 26730738 PMC4701484

[29] Van OJ, Voorrips R. JoinMap(R) 3.0. Software for the calculation of genetic linkage maps. Plant Research International, Wageningen 2001.

[30] Kosambi D. The estimation of map distances from recombination values. Ann Eugen. 1944;12:172–5.

[31] Liu K, Muse SV. PowerMarker: an integrated analysis environment for genetic marker analysis Oxford Univ Press; 2005. 10.1093/bioinformatics/bti282 15705655

[32] Nei M. Analysis of Gene Diversity in Subdivided Populations. Proc Natl Acad Sci U S A. 1973;70(12):3321–3. 4519626 10.1073/pnas.70.12.3321 PMC427228

[33] Tamura K, Dudley J, Nei M, Kumar S. MEGA4: Molecular Evolutionary Genetics Analysis (MEGA) Software Version 4.0. Mol Biol Evol. 2007;24(8):1596–9. 17488738 10.1093/molbev/msm092

[34] Temnykh S, DeClerck G, Lukashova A, Lipovich L, Cartinhour S, McCouch S. Computational and experimental analysis of microsatellites in rice (*Oryza sativa* L.): frequency, length variation, transposon associations, and genetic marker potential Genome Res. 2001;11:1441–52. 11483586 10.1101/gr.184001 PMC311097

[35] Wang Q, Fang L, Chen J, Hu Y, Si Z, Wang S, et al Genome-Wide Mining, Characterization, and Development of Microsatellite Markers in *Gossypium* Species. Scientific Reports. 2015;5:10638 10.1038/srep10638 26030481 PMC4650602

[36] Shi J, Huang S, Zhan J, Yu J, Wang X, Hua W, et al Genome-wide microsatellite characterization and marker development in the sequenced *Brassica* crop species. DNA Res. 2014;21(1):53–68. 10.1093/dnares/dst040 24130371 PMC3925394

[37] Sharma PC, Grover A, Kahl G. Mining microsatellites in eukaryotic genomes. Trends Biotechnol. 2007;25(11):490–8. 17945369 10.1016/j.tibtech.2007.07.013

[38] Sonah H, Deshmukh RK, Sharma A, Singh VP, Gupta DK, Gacche RN, et al Genome-wide distribution and organization of microsatellites in plants: an insight into marker development in *Brachypodium*. PLoS ONE. 2011;6(6):e21298 10.1371/journal.pone.0021298 21713003 PMC3119692

[39] Yasodha R. Characterization of microsatellites in the tribe Bambuseae. Gene Conserve. 2011;(39):51–64.

[40] Pandey G, Misra G, Kumari K, Gupta S, Parida SK, Chattopadhyay D, et al Genome-Wide Development and Use of Microsatellite Markers for Large-Scale Genotyping Applications in Foxtail Millet [*Setaria italica* (L.)]. DNA Res. 2013;20(2):197–207. 10.1093/dnares/dst002 23382459 PMC3628449

[41] Cheng X, Xu J, Xia S, Gu J, Yang Y, Fu J, et al Development and genetic mapping of microsatellite markers from genome survey sequences in *Brassica napus*. Theor Appl Genet. 2009;118(6):1121–31. 10.1007/s00122-009-0967-8 19190889

[42] Li H, Chen X, Yang Y, Xu J, Gu J, Fu J, et al Development and genetic mapping of microsatellite markers from whole genome shotgun sequences in *Brassica oleracea*. Mol Breeding. 2011;28(4):585–96.

[43] Pandey MK, Upadhyaya HD, Rathore A, Vadez V, Sheshshayee M, Sriswathi M, et al Genomewide Association Studies for 50 Agronomic Traits in Peanut Using the ‘Reference Set’Comprising 300 Genotypes from 48 Countries of the Semi-Arid Tropics of the World. PLoS ONE. 2014;9(8):e105228 10.1371/journal.pone.0105228 25140620 PMC4139351

[44] Jiang H, Huang L, Ren X, Chen Y, Zhou X, Xia Y, et al Diversity characterization and association analysis of agronomic traits in a Chinese peanut (*Arachis hypogaea* L.) mini-core collection. J Integr Plant Biol. 2014;56(2):159–69. 10.1111/jipb.12132 24237710

[45] Wang ML, Sukumaran S, Barkley NA, Chen Z, Chen CY, Guo B, et al Population structure and marker-trait association analysis of the US peanut (*Arachis hypogaea* L.) mini-core collection. Theor Appl Genet. 2011;123(8):1307–17. 10.1007/s00122-011-1668-7 21822942

[46] Li H, Younas M, Wang X, Li X, Chen L, Zhao B, et al Development of a core set of single-locus SSR markers for allotetraploid rapeseed (*Brassica napus* L.). Theor Appl Genet. 2013;126:937–47. 10.1007/s00122-012-2027-z 23238763

